# Delayed gastric emptying after laparoscopic pancreaticoduodenectomy: a single-center experience of 827 cases

**DOI:** 10.1186/s12893-024-02447-7

**Published:** 2024-05-11

**Authors:** Lingwei Meng, Jun Li, Guoqing Ouyang, Yongbin Li, Yunqiang Cai, Zhong Wu, Bing Peng

**Affiliations:** 1 Division of Pancreatic Surgery, Department of General Surgery, West China Hospital, Sichuan University, No. 37, Guoxue Alley, Chengdu, 610041 Sichuan Province China; 2 Departments of General Surgery, Chengdu Shangjin Nanfu Hospital, Chengdu, Sichuan Province China

**Keywords:** Delayed gastric emptying, Laparoscopic pancreaticoduodenectomy, Major complications, Pancreatic fistula

## Abstract

**Background:**

Delayed gastric emptying (DGE) commonly occurs after pancreaticoduodenectomy (PD). Risk factors for DGE have been reported in open PD but are rarely reported in laparoscopic PD (LPD). This study was designed to evaluate the perioperative risk factors for DGE and secondary DGE after LPD in a single center.

**Methods:**

This retrospective cohort study included patients who underwent LPD between October 2014 and April 2023. Demographic data, preoperative, intraoperative, and postoperative data were collected. The risk factors for DGE and secondary DGE were analyzed.

**Results:**

A total of 827 consecutive patients underwent LPD. One hundred and forty-two patients (17.2%) developed DGE of any type. Sixty-five patients (7.9%) had type A, 62 (7.5%) had type B, and the remaining 15 (1.8%) had type C DGE. Preoperative biliary drainage (*p* = 0.032), blood loss (*p* = 0.014), and 90-day any major complication with Dindo-Clavien score ≥ III (*p* < 0.001) were independent significant risk factors for DGE. Seventy-six (53.5%) patients were diagnosed with primary DGE, whereas 66 (46.5%) patients had DGE secondary to concomitant complications. Higher body mass index, soft pancreatic texture, and perioperative transfusion were independent risk factors for secondary DGE. Hospital stay and drainage tube removal time were significantly longer in the DGE and secondary DGE groups.

**Conclusion:**

Identifying patients at an increased risk of DGE and secondary DGE can be used to intervene earlier, avoid potential risk factors, and make more informed clinical decisions to shorten the duration of perioperative management.

## Introduction

Pancreaticoduodenectomy (PD) is the cornerstone treatment for pancreatic head and periampullary pathologies [1]. Despite significant improvements in surgical techniques and perioperative care over the past few decades, PD remains associated with high morbidity and mortality [2, 3]. Delayed gastric emptying (DGE) is one of the most common complications of PD, with an incidence rate ranging from 19 to 57% [4, 5]. It is rarely life-threatening but is associated with delayed oral intake, prolonged hospital stay, and increased total cost of hospitalization [5–7]. Several studies have focused on the risk factors of DGE, such as the preservation of the pylorus or the route of the gastric reconstruction loop [5, 7–10]. Similarly, the existence of primary and secondary causes of DGE has been proposed [11, 12]. However, these findings have been controversial.

Laparoscopic PD (LPD) was introduced in 1994 [13]. Over the past decade, an increasing number of studies have affirmed its safety, feasibility, and acceptable oncological outcomes [14, 15]. LPD has become the preferred surgical procedure in a few large pancreatic surgery centers [16–18]. However, few reports have analyzed the risk factors for DGE after LPD. This study aimed to characterize DGE and secondary DGE after LPD and analyze their specific risk factors and impact on the clinical postoperative course.

## Methods

### Patient selection

This single-center retrospective comparative study was performed at the Division of Pancreatic Surgery, Department of General Surgery, Huaxi Hospital, Sichuan University, Sichuan Province, China, between October 2010 and April 2023. The study was approved by the Ethics Committee of Sichuan University and was conducted in accordance with The Code of Ethics of the World Medical Association (Declaration of Helsinki). Informed consent was obtained from all patients. The privacy rights of participants were always observed. We excluded patients (1) undergoing LPD at other centers, (2) transitioning to open surgery, (3) undergoing total pancreatectomy as a change in the surgical procedure, and (4) whose data were not systematically collected. The analysis included 827 consecutive patients who underwent LPD. Medical history, laboratory values, perioperative characteristics, postoperative outcomes, complications, and mortality were recorded.

### Perioperative surveillance

Routinely monitored amylase in abdominal drainage fluid at 1, 3, and 5 days after surgery. Abdominal CT will be performed on the third day after surgery. The abdominal drainage tubes were removed after excluding pancreatic leakage, biliary leakage, gastrointestinal leakage, and abdominal fluid accumulation. The nasogastric tube (NGT) was placed before surgery and removed 1 or 2 days after surgery, and oral intake was advanced as tolerated. DGE was defined and graded according to the ISGPS consensus definition [4]. Patients who developed DGE were analyzed separately, and the risk factors for DGE were explored. Those with DGE were subsequently divided into primary and secondary DGE (pDGE and sDGE). sDGE was defined as a DGE occurring contemporarily or immediately afterward (< 5 days), with the development of another complication, including clinically relevant postoperative pancreatic fistula (CR-POPF, including POPF of grades B and C), post-pancreatectomy hemorrhage (PPH), chyle leak, biliary leak, and abdominal infection. DGE was otherwise defined as “primary” (pDGE). Other pancreas-specific complications, including POPF [19], PPH [20], and chyle leak [21], were defined according to the ISGPS classifications. Biliary leakage was defined according to the International Study Group for Liver Surgery classification [22]. Major complications were defined as a Dindo-Clavien grade ≥ III [23]. Abdominal infection was defined as the development of chills, fever, abdominal distension, and intestinal paralysis that lasted for more than 24 h after the third postoperative day. A significant increase in white blood cell count, hypoproteinemia, anemia on laboratory examination, fluid accumulation in the abdominal cavity on computed tomography (CT), and purulent aspiration fluid with bacteria confirmed the diagnosis. Reoperation was defined as a secondary operation due to severe complications within 90 days of LPD. Patients were discharged when oral intake and moderate activity were tolerated without any abnormal postoperative complications or laboratory findings.

### Surgical procedures

Surgical procedures were described in detail in our previous study [24]. The type of reconstruction procedure was child procedure, namely pancreaticojejunostomy, hepatico-jejunostomies anastomosis, and Roux-en-Y reconstruction of gastrojejunostomy or duodenojejunal anastomosis. Laparoscopic pancreaticoduodenectomy began with hand-assisted LPD, switched to total LPD, and gradually progressed to laparoscopic pylorus-preserving pancreaticoduodenectomy (LPPPD), considering that LPD is a challenging operation for most surgeons. Furthermore, our previous study indicated that no significant differences were observed among these three operation types in terms of intraoperative parameters or postoperative complications [24]. Therefore, hand-assisted LPD, total LPD, and LPPPD were regarded as homogeneous LPD methods in the analysis of the perioperative risk factors of DGE. Three to four abdominal drainage tubes will be placed inside the abdominal cavity to monitor postoperative complications.

### Statistical analysis

Statistical analysis of the variables was performed using SPSS software, version 23.

Continuous variables were compared using Student’s t-test or the Mann-Whitney U test. The chi-square test and Fisher’s exact test were used to compare categorical variables. Statistical significance was set at *P* < 0.05. Variables with *P* < 0.05 underwent multivariate analyses. Logistic regression was performed for the multivariate analysis to determine the main independent risk factors for DGE and sDGE. The independent risk factors for the variables are expressed as odds ratios (OR) with their 95% confidence intervals (CI).

## Results

### Prevalence of delayed gastric emptying

Of the 827 patients included in the study, 142 patients (17.2%) developed DGE of any grade. Sixty-five patients (7.9%) had grade A, 62 (7.5%) had grade B, and the remaining 15 (1.8%) had grade C. After univariate analysis, artificial vascular replacement (*p* < 0.001), preoperative jaundice (*p* = 0.049), preoperative biliary drainage (*p* = 0.011), longer operative time (*p* = 0.014), higher estimated blood loss volume (*p* = 0.001), CR-POPF (*p* < 0.001), PPH (*p* = 0.001), 90-day reoperation (*p* = 0.018), 90-day any major complication with Dindo-Clavien score ≥III (*p* < 0.001), biliary leak (*p* < 0.001), abdominal infection (*p* < 0.001), and pulmonary infection (*p* < 0.001) were significantly more frequent in patients with DGE than in patients without DGE. The demographics, preoperative characteristics, intraoperative findings, and postoperative outcomes are shown in Tables 1 and 2. In multivariate analysis, preoperative biliary drainage (*p* = 0.032), higher estimated blood loss volume (*p* = 0.014), and 90-day any major complication with Dindo-Clavien score ≥III (*p* < 0.001) were independent significant risk factors for DGE. Further details of the postoperative outcomes are listed in Table 3. There was a statistically significant (*p* < 0.001) increase in the length of hospital stay and removal time of the drainage tube in patients with DGE grades A, B, and C versus those with no DGE.

**Table 1:** Univariate analysis of clinical and intraoperative factors associated with the development of delayed gastric emptying. (*N* = 827)

	DGE *N* = 142 (17.2%)	No DGE *N* = 685(82.8%)	*P* value
Gender (Male/ Female)	62/80	292/393	0.821
Age (years, median, IQR)	63(55–70)	62(52–68)	0.059
BMI (kg/m^2^, median, IQR)	22.0(20.3–23.9)	22.2(20.2–24.0)	0.763
Hypertension	33(23.2%)	115(16.8%)	0.068
Diabetes mellitus	19(13.4%)	81(11.8%)	0.605
Smoking history	40(28.2%)	165(24.1%)	0.305
History of alcohol intake	21(14.8%)	102(14.9%)	0.975
History of abdominal surgery History of gastrointestinal surgery	12(8.5%) 3(2.1%)	79(11.5%) 14(2.0%)	0.285 1.000
Preoperative jaundice	54(38.0%)	203(29.6%)	0.049
Preoperative biliary drainage	36(25.4%)	112(16.4%)	0.011
Tumor locations			
Bile duct Pancreas Ampulla Duodenum	41(28.9%) 65(45.8%) 21(14.8%) **15(10.5%)**	179(26.1) 340(49.6) 100(14.6) **66(9.7%)**	0.501 0.402 0.953 0.735
Indications for surgery			
Benign/ Malignant	37/105	213/472	0.234
ASA score ≥ 3	54(38.0%)	235(34.3%)	0.335
Pancreatic texture(soft/hard)	61/81	331/354	0.244
Pylorus preserving	132(93.0%)	651(95.0%)	0.315
Combined organ resection	6(4.2%)	20(2.9%)	0.584
Vascular resection and reconstruction Side wall End to end Vascular grafts	26(18.3%) 7(4.9%) 5(3.5%) 14(9.9%)	89(13.0%) 65(9.5%) 17(2.5%) 7(1.0%)	0.096 0.079 0.679 < 0.001
Perioperative transfusion	21(14.8%)	90(13.1%)	0.604
Blood loss (ml, median, IQR)	175.0(100.0-365.0)	120.0(100.0-200.0)	0.001
Operative time (min, median, IQR))	370.0 (283.8-441.3)	330.0(273.0-401.5)	0.014

ASA, American Society of Anesthesiologists; BMI, Body Mass Index

**Table 2:** Postoperative outcomes among patients with and without DGE

	DGE *N* = 142 (17.2%)	No DGE *N* = 685(82.8%)	*P* value
Clinically-relevant pancreatic fistula Grade B Grade C	33(23.2%) 27(19.0%) 6(4.2%)	70(10.2%) 60(8.7%) 10(1.5%)	< 0.001 < 0.001 0.065
Intestinal obstruction	6(4.2%)	12(1.8%)	0.066
Postpancreatectomy hemorrhage Re-operation Conservative	16(11.3%) 10(7.0%) 6(4.2%)	29(4.2%) 22(3.2%) 7(1.0%)	0.001 0.031 0.015
Biliary leak	12(8.5%)	15(2.2%)	< 0.001
Chyle leak	19(13.4%)	63(9.2%)	0.129
Wound infection	5(3.5%)	12(1.8%)	0.304
Abdominal infection	30(21.1%)	54(7.9%)	< 0.001
Pulmonary infection	24(16.9%)	49(7.2%)	< 0.001
Death within 90 days	3(2.1%)	8(1.2%)	0.623
90d-reoperation	14(9.9%)	33(4.8%)	0.018
90d any major complication	47(33.1%)	96(14.0%)	< 0.001
Removal time of drainage tubes (days, median, IQR)	11(8–20)	8(7–12)	< 0.001
Length of hospital stay (days, median, IQR))	18(14–25)	13(10–17)	< 0.001

90d any major complication: Dindo-Clavien score ≥III

**Table 3:** Multivariable analysis of perioperative risk factors for DGE (*n* = 827)

Variables	B	SE	Wals	*P* value	OR	95% CI
Preoperative biliary drainage	0.485	0.226	4.597	0.032	1.624	1.043–2.530
Blood loss (ml)	0.001	0.000	5.993	0.014	1.001	1.000-1.002
90d any major complication	1.066	0.212	25.342	< 0.001	2.904	1.918–4.399

90d any major complication: Dindo-Clavien score≥III

### Primary versus secondary DGE

Of the 142 patients complicated by DGE, 76 (54.2%) were diagnosed with pDGE, whereas 66 (45.8%) patients had DGE secondary to concomitant complications. There were no significant differences between pDGE and sDGE in terms of age, sex, ASA score, presence of diabetes mellitus and hypertension, smoking history, history of alcohol intake, preoperative jaundice, preoperative biliary drainage, history of abdominal or gastrointestinal surgery, operative time, organ or vascular resection, or estimated blood loss. Type C DGE was significantly more common in sDGE (*p* = 0.001). The lengths of hospital stay and drainage tube removal time were significantly shorter in the pDGE group. Tables 4 and 5 present detailed comparisons of the pDGE and sDGE subgroups.

**Table 4:** Univariate analysis of clinical and intraoperative factors between pDGE and sDGE

	pDGE *N* = 76 (53.5%)	sDGE *N* = 66(46.5%)	*p*
Gender (Male/ Female)	38/38	42/24	0.102
Age (years, median, IQR)	63(54–70)	62(55–70)	0.731
BMI (kg/m^2^, median, IQR)	21.3(19.6–23.3)	22.8(21.2–24.5)	0.003
Hypertension	17(22.4%)	16(24.2%)	0.792
Diabetes mellitus	11(14.5%)	8(12.1%)	0.681
Smoking history	20(26.3%)	20(30.3%)	0.598
History of alcohol intake	9(11.8%)	12(15.8%)	0.288
History of abdominal surgery	5(6.6%)	7(10.6%)	0.390
History of gastrointestinal surgery	2(2.6%)	1(1.3%)	1.000
Preoperative jaundice	31(40.8%)	23(34.8%)	0.467
Preoperative biliary drainage	20(26.3%)	16(24.2%)	0.777
Tumor location			
Bile duct Pancreas Ampulla Duodenum	23(30.3%) 36(47.4%) 6(7.9%) **11(14.5%)**	18(27.3%) 29(43.9%) 9(13.6%) **10(15.2%)**	0.818 0.682 0.267 0.910
Indications for surgery			
Benign/ Malignant	21/55	16/50	0.646
ASA score ≥ 3	33(43.4%)	23(34.8%)	0.297
Pancreatic texture(soft/hard)	26/50	35/31	0.024
Pylorus preserving	72(94.7%)	60(90.9%)	0.575
Combined organ resection	5(6.6%)	1(1.5%)	0.281
Vascular resection and reconstruction Side wall End to end Vascular grafts	14(18.4%) 7(9.2%) 3(3.9%) 4(5.3%)	12(15.2%) 7(10.6%) 2(2.6%) 3(4.5%)	0.971 0.781 1.000 1.000
Perioperative transfusion	7(9.2%)	14(21.2%)	0.044
Blood loss (ml, median, IQR)	365(300–439)	370(267–446)	0.797
Operative time (min, median, IQR))	150(100–350)	200(100–385)	0.163

ASA, American Society of Anesthesiologists; BMI, Body Mass Index; Secondary DGE (sDGE); primary DGE (pDGE)

**Table 5:** Comparison of postoperative outcome between primary and secondary DGE

	pDGE *N* = 76 (53.5%)	sDGE *N* = 66(46.5%)	*P* value
DGE ISGPS grade			
A B C	36(47.4%) 38(50.0%) 2(2.6%)	29(43.9%) 24(36.4%) 13(19.7%)	0.682 0.102 0.001
Intestinal obstruction	1(1.3%)	5(7.6%)	0.152
Wound infection	0(0.0%)	5(7.6%)	0.047
Pulmonary infection	12(15.8%)	12(18.2%)	0.704
Death within 90 days	0(0.0%)	3(4.5%)	0.196
90d-reoperation	0(0.0%)	11(16.7%)	0.001
90d any major complication	0(0.0%)	47(71.2%)	< 0.001
Removal time of drainage tubes (days, median, IQR)	9(7–11)	20(12–25)	< 0.001
Length of hospital stay (days, median, IQR))	16(13–19)	23(17–35)	< 0.001

90d any major complication, Dindo-Clavien score ≥III; sDGE, secondary DGE; pDGE, primary DGE

### Risk factors for sDGE

In the subgroup analysis of sDGE predictors, higher body mass index (BMI) (*p* = 0.036), soft pancreatic texture (*p* = 0.002), and perioperative transfusion (*p* = 0.004) were independent risk factors with statistical significance in the multivariate analysis (Table 6).

**Table 6:** Multivariable analysis of perioperative risk factors for secondary DGE

Variables	B	SE	Wals	*P* value	OR	95% CI
BMI	0.145	0.069	4.398	0.036	1.156	1.010–1.324
Soft gland texture	1.241	0.397	9.781	0.002	3.457	1.589–7.522
Perioperative transfusion	1.607	0.557	8.334	0.004	4.988	1.675–14.853

BMI, Body Mass Index

## Discussion

LPD is one of the most technically challenging surgical procedures, involving complicated dissection and reconstruction. The primary concern is the high incidence of postoperative morbidity and mortality. Improved surgical techniques and technologies, together with an increase in operative volume and surgeon experience in high-volume centers, have reduced the incidence of postoperative complications and improved overall survival [17, 25, 26]. Recently, the hospital mortality rate after LPD has notably decreased to less than 6% [16–18]. This study shows the 90-day mortality rate was 1.3%, similar to previous reports. However, the incidence of postoperative major complications remains high [17, 18]. DGE remains a particular concern for pancreatic surgeons, even in high-volume centers, because of its high incidence. In Wang et al.’s [17] multicenter study, which included 1029 patients, major complications occurred in 49.66% of patients, and 16.72% experienced DGE. Another study [14] reported DGE (grade B/C) in 9% of the LPD group. Li et al. [27] reported that DGE occurred in 33.2% of patients, with grades B and C occurring in 21.6%. In our study, the 90-day overall major complication rate was 17.3%, DGE was noted in 17.2% of patients, and 9.3% developed DGE grade B/C. In a single-center study by Song et al. [15]. that included 500 patients who underwent LPD, the severe complication rate was 4.8%; moreover, 2.4% of patients had DGE. This variation in incidence may be explained by different institutional practices, with a tendency to delay nasogastric tube removal and oral feeding.

In this retrospective study, we investigated the risk factors of DGE in 827 patients who underwent LPD and found that the need for preoperative biliary drainage, higher blood loss, and 90-day any major complication (Dindo-Clavien ≥ III) were significant independent risk factors for DGE occurrence. Preoperative factors, including preoperative biliary drainage, but not preoperative jaundice, were associated with increased DGE in this study, which showed inconsistent results regarding demographic characteristics that may contribute to DGE, including age, sex, smoking history, and surgical indications [5, 12]. It is speculated that patients requiring preoperative biliary drainage may have more blood loss, leading to higher rates of a difficult postoperative course and being more in line with the diagnosis of DGE. Blood loss, an operative factor, was associated with increased DGE in our study, consistent with previous studies [27–29] and our preoperative factors. Several studies have reported that postoperative complications, including hemorrhage, CR-POPF, and abdominal infection, significantly influence the incidence of DGE after surgery [5, 9, 11, 12, 27, 29]. Further, our analysis showed that the presence of major complications (Dindo-Clavien ≥ III) was significantly associated with increased DGE occurrence, consistent with previous studies [7, 27]. This indicates that severe postoperative complications have a significant impact on postoperative DGE rather than being limited to severe abdominal complications. Therefore, the management of patients with DGE should not only focus on managing DGE and abdominal complications but also pay more attention to the improvement of the overall state, such as pulmonary infection and incision infections.

Furthermore, we divided DGE into pDGE and sDGE and explored the risk factors for sDGE. We found that higher BMI, soft pancreatic texture, and perioperative transfusion were independent risk factors in the multivariate analysis, which was consistent with the distinction between pDGE and sDGE and has already been proposed in other studies [11, 12, 30], although a consensus on such characterization has not been reached. As expected, most predictors, including higher BMI and soft pancreatic texture of sDGE, largely overlapped with those related to CR-POPF. Therefore, patients at a higher risk of sDGE could benefit from specific pathways in the early postoperative phase, such as those adopted to avoid CR-POPF [11, 29]. Perioperative transfusion may be associated with a poorer general condition, longer surgical time, and intraoperative bleeding, which further increases the risk of postoperative complications and leads to a higher incidence of sDGE. Meanwhile, the incidence of sDGE was higher than that of pDGE compared with other studies, which may be highly related to the fewer secondary factors listed; however, this did not affect the consistency of our analysis results with others.

In conclusion, preoperative biliary drainage, blood loss, and 90-day any major complication with a Dindo-Clavien score ≥III were strongly associated with DGE after LPD. Furthermore, higher BMI, soft pancreatic texture, and perioperative transfusion were independent risk factors for sDGE. Identifying patients at increased risk for DGE and sDGE can be used to intervene earlier, avoid potential risk factors, and make more informed clinical decisions to shorten the duration of perioperative management.

## Data Availability

All data generated or analysed during this study are included in this published article. The datasets used and/or analysed during the current study are available from the corresponding author upon reasonable request.

## Abbreviations

DGE: Delayed gastric emptying
PD: Pancreaticoduodenectomy
LPD: Laparoscopic pancreaticoduodenectomy
BMI: Body Mass Index
pDGE: Primary delayed gastric emptying
sDGE: Secondary delayed gastric emptying
CR-POPF: Clinically relevant postoperative pancreatic fistula
PPH: Post-pancreatectomy hemorrhage
ISGPS: International Study Group for Pancreatic Surgery
CT: Computed tomography
OR: Odds ratios
CI: Confidence intervals
ASA: American Society of Anesthesiologists
